# Febrile Seizures in Children in a Low-Resource Setting: A Hospital-Based Cross-Sectional Study from Eastern Libya

**DOI:** 10.1055/s-0046-1817789

**Published:** 2026-03-24

**Authors:** Zinelabedin Mohamed, Mohamed Baklola, Aisha Ahmeedah, Marzouga Ahmeedah, Ahmed R. Abdullah, Amani Mohammed, Mohammed Omar, Fatimah Jabullah, Dua Amir, Ibrahim Elsayed

**Affiliations:** 1 Faculty of Medicine, Tobruk University, Tobruk, Libya; 2 Faculty of Medicine, Mansoura University, Mansoura, Egypt; 3 Biochemistry and Molecular Biology Department, Faculty of Pharmacy, Al-Azhar University, Cairo, Egypt

**Keywords:** seizures, febrile, epilepsy, children

## Abstract

**Background:**

Febrile seizures (FSs) are the most common seizure disorder in early childhood, affecting approximately 2 to 5% of children under 5 years of age. Although generally benign, FSs cause significant parental distress and health care utilization and are commonly triggered by childhood infections. Data from low-resource health care settings remain limited. This study aimed to assess the hospital-based prevalence, clinical characteristics, and factors associated with FSs among children in eastern Libya.

**Methods:**

This retrospective cross-sectional study was conducted from August 1, 2022, to August 31, 2023, at Tobruk Medical Center, a tertiary referral hospital in eastern Libya. A total of 2,604 pediatric admissions of children aged 6 to 60 months were screened, and 140 children admitted with FSs were included. Demographic, clinical, and laboratory data were extracted from medical records and caregiver interviews. Statistical analyses were performed using SPSS version 27.

**Results:**

The hospital-based prevalence of FSs was 5.7%, with equal sex distribution. Simple FSs accounted for 87.9% of cases, while complex FSs comprised 12.1%. The median age at seizure onset was 18 months. A family history of epilepsy was significantly more common among children with complex FSs than among those with simple FSs (29.4% vs. 6.5%, *p* = 0.009). Upper respiratory tract infections were the most frequent precipitating cause of fever (72.8%). Children with complex FSs experienced more frequent seizures during a single febrile illness and had higher white blood cell counts.

**Conclusion:**

In this hospital-based study from a resource-limited setting, FSs, predominantly simple FSs, were common among young children. A family history of epilepsy and elevated white blood cell counts were associated with complex FSs, highlighting the importance of risk stratification and context-appropriate management in low-resource health care environments.

## Introduction

Febrile seizures (FSs) are the most common type of seizure in children, affecting 2 to 5% of the pediatric population under 5 years of age, with peak incidence occurring between 18 and 24 months. 1 2 They account for a significant portion of pediatric neurological cases, presenting a substantial challenge in both clinical management and parental anxiety. 3 The International League Against Epilepsy defines FSs as seizures associated with a febrile illness in children aged 6 months to 5 years, without evidence of central nervous system infection, metabolic disturbance, or a history of afebrile seizures. 3 Although FSs are generally considered benign, their sudden onset and dramatic presentation underscore the importance of understanding their epidemiology and clinical characteristics to optimize pediatric care. 4

The etiology of FSs remains multifactorial, with genetic, environmental, and immunological factors playing roles in their development. 5 6 A positive family history of FSs or epilepsy has been consistently identified as an important risk factor, supporting a genetic predisposition. FSs are most commonly precipitated by acute childhood infections, particularly those involving the upper respiratory and gastrointestinal systems. 5 Among these, upper respiratory tract infections are frequently reported as the leading trigger, followed by gastrointestinal infections and other febrile illnesses such as urinary tract infections and acute otitis media. 7

Clinically, FSs are categorized as simple FSs (SFS) or complex FSs (CFS). 1 SFS, which are more common, last less than 15 minutes, are generalized in nature, and do not recur within 24 hours. 1 2 Children with CFS have a relatively higher risk of subsequent epilepsy compared with those experiencing SFS. 8 Febrile status epilepticus, defined as a seizure lasting more than 30 minutes, represents a severe but uncommon manifestation and is associated with increased morbidity. 8 Despite the favorable long-term prognosis in most cases, the risk of recurrence and potential progression to more severe seizure phenotypes necessitate careful clinical assessment and follow-up. 9

Several regional and international studies have examined the prevalence and clinical characteristics of FSs. In Libya, a hospital-based study conducted in 2018 reported a prevalence of 5.7% among children aged 6 months to 5 years, with a predominance of SFS and a higher frequency among males. 10 Similarly, a study from Bayda City in eastern Libya found that most FSs were simple in nature and were commonly associated with respiratory tract infections. 11 While these studies have contributed valuable insights, available data from eastern Libya remain limited, and few reports have examined FSs within the context of resource-constrained health care settings.

Against this background, the present study aims to provide updated hospital-based data on the prevalence and clinical characteristics of FSs among children in eastern Libya and to identify factors associated with CFS. By situating these findings within a low-resource health care environment, this study seeks to enhance understanding of regional epidemiological patterns and inform context-appropriate clinical management strategies for FSs in similar settings.

## Methods

### Study Design and Period

This was a retrospective cross-sectional study conducted in the Department of Pediatrics at Tobruk Medical Center (TMC), the sole tertiary referral hospital with an inpatient pediatric ward in eastern Libya. TMC provides pediatric care to children from Tobruk city and surrounding regions, with a catchment area extending from Emsaed at the eastern border to Al-Tamimi city west of Tobruk, serving an estimated population of approximately 18,000 children aged 0 to 4 years. 12

The study was conducted over a 13-month period, from August 1, 2022, to August 31, 2023, and included children aged 6 to 60 months who were admitted to the pediatric ward from the emergency department with a diagnosis of FSs. In our institution, all children presenting with FSs, both simple and complex, are routinely admitted for observation and evaluation according to local hospital policy, which reflects limited outpatient follow-up capacity and the absence of pediatric neurology consultation services.

In line with best practices for reporting observational studies, this study adhered to the STROCSS (Strengthening the Reporting of Cohort, Cross-Sectional, and Case-Control Studies in Surgery) criteria to ensure methodological transparency and consistency. 13

### Context of the Setting

This study was carried out in a resource-limited hospital environment. At TMC's Pediatric Department, staffing and infrastructure limitations significantly influence clinical care. Only one nurse is available per shift to care for all patients in the pediatric intensive care unit. Additionally, the facility experiences shortages of respiratory support equipment and has limited access to advanced diagnostic tools.

Specialty consultation services, including pediatric neurology, infectious disease, and pediatric intensive care specialists, are not routinely available, which presents additional challenges in the management of acute neurological conditions such as FSs. These constraints necessitate reliance on clinical judgment and basic investigations and contribute to lower thresholds for hospital admission and observation, particularly for young children with seizures.

### Sample Size Calculation

The initial sample size was calculated using OpenEpi Version 3.01 based on an expected prevalence of FSs in pediatric populations, with a 5% margin of error, 95% confidence level, and 80% study power. This yielded a minimum required sample size of 364 children.

However, all eligible children presenting during the study period were included, resulting in a total of 2,604 pediatric admissions being screened and 140 confirmed cases of FSs. Including all eligible cases enhanced the precision of prevalence estimates and increased the statistical power of comparative analyses, particularly between SFS and CFS groups.

### Study Population

The study population consisted of children aged 6 to 60 months who were admitted to the pediatric ward with FSs. Inclusion criteria were a documented body temperature of ≥ 38°C at presentation or reported by caregivers, and a seizure episode not attributable to central nervous system infection, metabolic disturbance, trauma, or intoxication. Only children experiencing their first episode of FS during the study period were included.

Children were excluded if they had a prior diagnosis of epilepsy, a history of afebrile seizures, known underlying neurological disorders, or seizures secondary to metabolic abnormalities. Patients with incomplete medical records or missing essential clinical or laboratory data were also excluded.

### Data Collection

Data were collected from hospital records and interviews with the children's parents or guardians. Demographic and clinical data included age, sex, body temperature, type of seizure (simple or complex), number of episodes, family history of FSs and epilepsy, and causes of fever. Fever triggers were categorized as upper respiratory tract infection, gastrointestinal infection, acute otitis media, urinary tract infection, and nonspecific or other causes. Laboratory data, including complete blood count, C-reactive protein (CRP), blood sugar, and serum electrolytes, were also recorded for each patient. Although international guidelines do not recommend routine laboratory testing for SFS, these investigations are commonly performed in our institution according to local practice to exclude serious bacterial infection, particularly in a low-resource setting with limited access to advanced diagnostics and subspecialty consultation.

### Statistical Analysis

All collected data were entered into Microsoft Excel and subsequently analyzed using IBM SPSS Statistics (Version 27). Descriptive statistics were used to summarize demographic and clinical characteristics. Continuous variables were tested for normality using the Kolmogorov–Smirnov and Shapiro–Wilk tests. Normally distributed data were expressed as means ± standard deviations, while nonnormally distributed data were presented as medians with interquartile ranges. Categorical variables were expressed as frequencies and percentages. Comparisons between SFS and CFS groups were performed using the chi-square test or Fisher's exact test for categorical variables, and Student's *t* -test or Mann–Whitney *U* test for continuous variables, as appropriate. A *p* -value of < 0.05 was considered statistically significant.

## Results

### Prevalence, Demographics, and Seizure Types

**Fig. 1:**
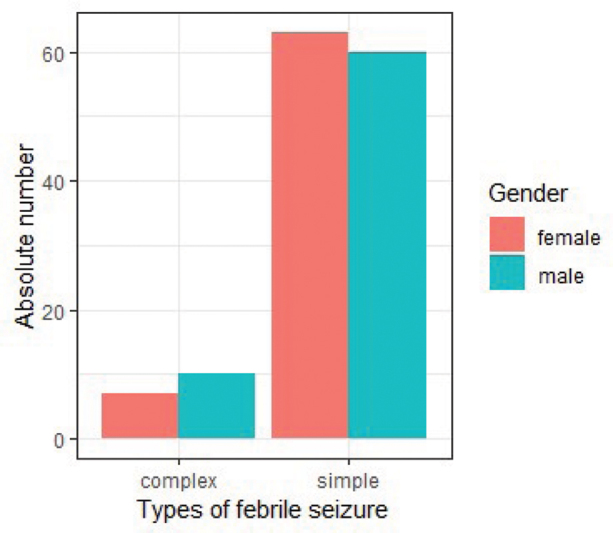
Gender distribution of children admitted with febrile seizures.

Among 2,604 pediatric admissions of children aged 6 to 60 months screened during the study period, 140 children were identified as having FSs, yielding a hospital-based prevalence of 5.7%. Of these, 70 (50%) were males and 70 (50%) were females, resulting in a male-to-female ratio of 1:1. The median age of children with FSs was 18 months, with a range of 4 to 60 months. SFS accounted for 87.9% (123 cases), while CFS comprised 12.1% (17 cases). The median age at seizure onset for SFS was 18 months (range: 13.00–29.50 months), and for CFS, it was 20 months (range: 12.00–35.00 months) ( Table 1 , Fig. 1 ).

**Table 1:** Sociodemographic data of children with febrile seizures

Clinical features		Complex febrile seizure ( *n* = 17)	Simple febrile seizure ( *n* = 123)	*p*
Gender	Female	7 (41.2)	63 (51.2)	0.61
	Male	10 (58.8)	60 (48.8)	
Age (mo)		20 (12–35)	18 (13–29.5)	0.63

Note: Data are expressed as *n* (%), median (interquartile range). Statistically significant at *p* < 0.05.

### Family History and Causes of Fever

**Fig. 2:**
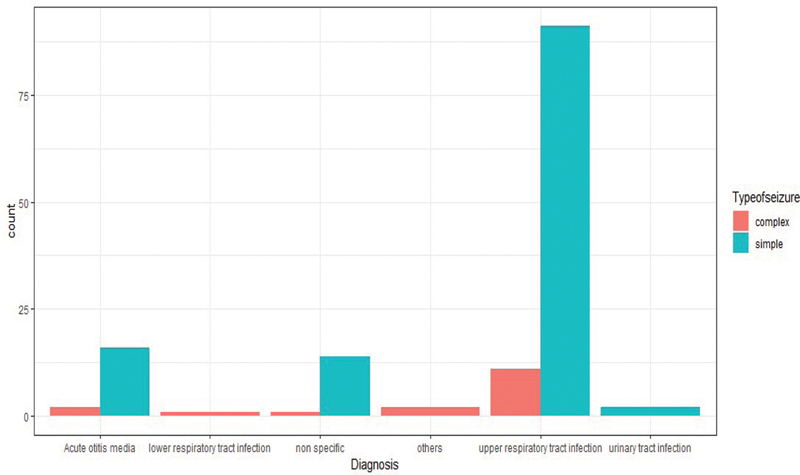
Possible causes of febrile seizures in children.

A family history of epilepsy was more common in the CFS group (29.4%) compared to the SFS group (6.5%) ( *p* = 0.009). Similarly, a family history of FSs was present in 41.2% of children with CFS and 35.8% with SFS, though this difference was not statistically significant ( *p* = 0.87). The most common cause of fever was upper respiratory tract infection, responsible for 72.8% of cases (102 children), followed by acute otitis media at 12.8% (18 children). These findings are summarized in Table 2 and illustrated in Fig. 2 .

**Table 2:** The association between febrile seizures and numerical clinical data

Clinical features		Complex febrile seizure ( *n* = 17)	Simple febrile seizure ( *n* = 123)	*p*
Family history of epilepsy	Absent	12 (70.6)	115 (93.5)	0.009 a
	Present	5 (29.4)	8 (6.5)	
Infection that triggered fever among children	Acute otitis media	2 (11.8)	16 (13)	< 0.001 a
	Lower respiratory tract infection	1 (5.9)	0 (0)	
	Nonspecific	1 (5.9)	14 (11.4)	
	Others	2 (11.8)	0 (0)	
	Upper respiratory tract infection	11 (64.6)	91 (74)	
	Urinary tract infection	0 (0)	2 (1.6)	
Development history	Delay	1 (5.9)	3 (2.4)	0.98
	Normal	16 (94.1)	120 (97.6)	
Family history of febrile seizure	No	10 (58.8)	79 (64.2)	0.87
	Yes	7 (41.2)	44 (35.8)	

Note: Data are expressed as *n* (%).

a Statistically significant at *p* < 0.05.

### Seizure Characteristics and Laboratory Findings

There was no significant difference in the median age at the first FS between the SFS and CFS groups ( *p* = 0.96). However, children with CFS experienced more frequent seizures during a single febrile illness ( *p* < 0.001) and had higher rates of recurrent FSs ( *p* = 0.01). Additionally, elevated white blood cell (WBC) counts were significantly associated with CFS ( *p* = 0.03), while other laboratory parameters, such as hemoglobin, platelet count, sodium, potassium, calcium, blood sugar, and CRP levels, did not show significant differences between the two groups ( Table 3 ).

**Table 3:** The association between febrile seizures and categorical clinical data

Clinical features	Complex febrile seizure ( *n* =17) Median (IQR)	Simple febrile seizure ( *n* = 123) Median (IQR)	*p*
Age at first episode of febrile seizure (mo)	18 (12–20)	17 (11–25.5)	0.96
Temperature at time of admission	39 (38–39)	38.7 (38–39.25)	0.92
Episode of recurrent febrile seizure	0 (0–2)	0 (0–0)	0.01 a
Seizure frequents in single febrile illness	2 (2–2)	1 (1–1)	< 0.001 a
Hb	11.1 (11–11.9)	11.1 (10.6–11.8)	0.53
PLT	303 (255–343)	306 (265–360.5)	0.71
WBC	14 (12.4–16.3)	12.4 (9.50–15.35)	0.03 a
Calcium levels	9.1 (8.6–9.95)	9.1 (8.65–9.70)	0.84
Na levels	134 (132.2–135)	135 (133.7–136.6)	0.12
K levels	4.1 (3.84–4.2)	4.1 (3.9–4.3)	0.88
Blood sugar levels	100 (68–106)	103.5 (93.5–116.5)	0.07
CRP levels	8.05 (1.6–11.1)	8.05 (2.99–24)	0.52

Abbreviations: CRP, C-reactive protein; Hb, hemoglobin; IQR, interquartile range; PLT, platelet; WBC, white blood cell.

Note: Data are expressed as median (IQR).

a Statistically significant at *p* < 0.05.

## Discussion

Our study found that the hospital-based prevalence of FSs among children admitted to TMC was 5.7%, which is comparable to findings from previous studies conducted in Libya and neighboring countries. A Libyan hospital-based study conducted in 2018 reported an identical prevalence of 5.7%, suggesting stability in the burden of FSs within this region over time. 10 It is important to note that this estimate reflects hospitalized cases and should not be interpreted as a community incidence rate. The prevalence observed in our study was lower than the 8.8% reported in an Egyptian family health center and slightly lower than the 6.8% reported in a community-based study from Saudi Arabia. 14 15 These differences may be explained by variations in study design, health care-seeking behavior, admission policies, and access to outpatient services across settings.

With respect to sex distribution, our study demonstrated an equal male-to-female ratio (1:1), which differs from several regional reports describing male predominance. For instance, earlier Libyan studies reported a male-to-female ratio of 1.35:1, while data from Saudi Arabia suggested a higher prevalence among females. 10 15 Such variability may reflect differences in sample size, referral patterns, cultural factors influencing health care utilization, or underlying genetic diversity rather than true biological differences.

The median age at first FS in our cohort was 18 months, with similar ages observed among children with SFS and CFS. This finding reinforces existing evidence that the second year of life represents the period of highest vulnerability to FSs, likely due to neurodevelopmental immaturity and heightened susceptibility to fever-related neuronal excitability. 10 11 A study from Saudi Arabia indicated that 40.9% of children experienced FSs between the ages of 12 and 16 months, similar to our findings. 15 Age remains one of the strongest predictors of FS onset, with younger children being more susceptible, as supported by literature across different countries. 14 15 16

In terms of seizure classification, SFS accounted for 87.9% of cases, while CFS comprised 12.1%. This pattern aligns with international literature indicating that the majority of FSs are simple and self-limiting. 10 14 Notably, a previous Libyan study reported a higher proportion of atypical or complex features, including prolonged seizures and recurrence within the same febrile illness. 11 These differences may reflect variations in admission thresholds, case definitions, or referral bias, particularly in resource-limited settings where clinicians may adopt a more cautious approach.

In terms of family history, 41.2% of children with CFS in our study had a family history of FSs, compared to 35.8% in the SFS group. This is consistent with findings from Saudi Arabia, where 31.8% of children had a family history of FSs. 15 A positive family history has been widely recognized as a significant risk factor for FSs, likely due to genetic predisposition. 17 Moreover, a family history of epilepsy was found in 29.4% of children with CFS, suggesting an elevated risk of future epileptic conditions in these patients. These findings underscore the importance of considering family medical history in the assessment and management of FSs, echoing the conclusions of research conducted globally. 18

Upper respiratory tract infections were the predominant trigger for FSs in our cohort, accounting for 72.8% of cases. This finding is consistent with regional studies from Libya and Egypt and reflects the high burden of viral respiratory infections in early childhood. 11 14 This is not surprising, as viral infections often cause rapid increases in body temperature, which can provoke FSs in susceptible children. 1 These observations reinforce the importance of effective fever recognition and management as a key preventive strategy, particularly in settings with limited access to emergency services.

A notable finding of this study was the significant association between elevated WBC counts and CFS. This suggests that children with CFS may experience a more pronounced inflammatory response or more severe underlying infection. Similar associations have been reported in other studies examining laboratory markers in FSs. 19 Although international guidelines do not recommend routine laboratory testing for SFS, our findings support the cautious use of basic investigations in selected cases, particularly in low-resource settings where advanced diagnostic tools and specialist consultations are not readily available. In contrast, other laboratory parameters, including electrolyte levels, hemoglobin, and CRP, were not significantly associated with seizure complexity, indicating limited predictive utility.

The findings of this study have practical implications for the management of FSs in resource-limited health care environments. Clear differentiation between SFS and CFS remains essential for guiding observation, investigation, and parental counseling. 20 In settings such as ours, where outpatient follow-up and subspecialty care are constrained, hospital admission and basic laboratory evaluation may represent pragmatic strategies to ensure patient safety. 21 Furthermore, identifying children with a family history of epilepsy or elevated inflammatory markers may help clinicians prioritize closer monitoring and targeted education for caregivers.

## Limitations

This study has several limitations that should be acknowledged. First, the single-center, hospital-based design may limit the generalizability of the findings to other regions or health care settings with different population characteristics, admission policies, or levels of resource availability. Because the study included only hospitalized children, the findings may overrepresent more severe cases and should not be extrapolated to community-based incidence or prevalence estimates. Second, the retrospective cross-sectional nature of the study, with data obtained from medical records and caregiver reports, may be subject to information and recall bias, particularly regarding family history of seizures and fever characteristics. Third, the use of routine laboratory investigations reflects local institutional practice in a resource-limited setting and may not be generalizable to settings where guideline-based selective testing is feasible.

Additionally, long-term outcomes, including seizure recurrence beyond the study period and subsequent development of epilepsy, were not assessed, limiting the ability to evaluate prognosis. Finally, although the overall sample size was large, the relatively small number of children with CFS reduced the statistical power to detect associations within this subgroup. Future studies employing multicenter designs, standardized management protocols, and prospective follow-up are warranted to address these limitations and enhance external validity.

## Conclusion

FSs are a common neurological condition in early childhood, with SFS representing the majority of cases. In this hospital-based study from eastern Libya, conducted within a resource-limited health care environment, upper respiratory tract infections were the most frequent precipitating factor for FSs. A family history of FSs or epilepsy and elevated WBC counts were significantly associated with CFS, which were also characterized by higher recurrence rates. Although FSs are generally benign, children presenting with complex features may require closer observation, particularly in settings with limited access to outpatient follow-up and subspecialty care. The findings of this study provide updated regional epidemiological data and highlight the practical challenges of FS management in low-resource settings. Further research with prospective, multicenter designs and long-term follow-up is needed to evaluate outcomes such as epilepsy risk and to develop context-appropriate management strategies for FSs in similar health care environments.

## References

[1] Tiwari A Meshram R J Kumar Singh R Febrile seizures in children: a review Cureus 2022 14 11 e31509 36540525 10.7759/cureus.31509 PMC9754740

[2] Syndi Seinfeld D O Pellock J M Recent research on febrile seizures: a reviewJ Neurol Neurophysiol 2013;4(165) 10.4172/2155-9562.1000165 PMC4220240 25383238

[3] Dubé C M Brewster A L Richichi C Zha Q Baram T Z Fever, febrile seizures and epilepsy Trends Neurosci 2007 30 10 490 496 17897728 10.1016/j.tins.2007.07.006 PMC2766556

[4] Kilic B Clinical Features and Evaluation in Terms of Prophylaxis of Patients with Febrile Seizures SiSli Etfal Hastanesi Tip Bulteni/The Medical Bulletin of Sisli Hospital 2019 10.14744/SEMB.2019.30633 PMC7192276 32377096

[5] Sawires R Buttery J Fahey M A review of febrile seizures: recent advances in understanding of febrile seizure pathophysiology and commonly implicated viral triggers Front Pediatr 2022 9 801321 35096712 10.3389/fped.2021.801321 PMC8793886

[6] Semple B D Dill L K O'Brien T J Immune challenges and seizures: how do early life insults influence epileptogenesis? Front Pharmacol 2020 11 2 32116690 10.3389/fphar.2020.00002 PMC7010861

[7] Mosili P Maikoo S Mabandla M V Qulu L The pathogenesis of fever-induced febrile seizures and its current state Neurosci Insights 2020 15 2.633105520956973E15 10.1177/2633105520956973 PMC7649866 33225279

[8] Berg A T Shinnar S Complex febrile seizures Epilepsia 1996 37 02 126 133 8635422 10.1111/j.1528-1157.1996.tb00003.x

[9] DeLorenzo R J Hauser W A Towne A R A prospective, population-based epidemiologic study of status epilepticus in Richmond, Virginia Neurology 1996 46 04 1029 1035 8780085 10.1212/wnl.46.4.1029

[10] DMajeed N SS ALRaziq N A Abdullah N I Febrile convulsion in Libyan children Mediterranean J Pharm Pharmaceut Sci 2023 3 02 19 26

[11] محمد اا, محمد شن.Demography and Risk Factors of Febrile Convulsion in North Eastern Part of Libya “2012.” 2017 1 84

[12] Libya: Districts, Major Cities & Urban Settlements - Population Statistics, Maps, Charts, Weather and Web InformationAccessed December 26, 2025 at:https://www.citypopulation.de/en/libya/cities/?cityid=1281

[13] STROCSS Group Mathew G Agha R Albrecht J STROCSS 2021: strengthening the reporting of cohort, cross-sectional and case-control studies in surgery Int J Surg 2021 96 106165 34774726 10.1016/j.ijsu.2021.106165

[14] Nancy B Hosnia R Soad S Hanaa S Frequency and characteristics of febrile convulsions among children in Belbeis family health centerZagazig Univ Med J 2019;0(00):0–0

[15] Alhumaidy M Al-Mulhim Y Alabdullah W Alshakhs F Husain M Alsubaie Z Prevalence and risk factors of febrile convulsions among infants and children in Saudi Arabia Int J Med Develop Count 2020 1520 1526

[16] Almojali A I Ahmed A E Bagha M Y Prognostic factors for epilepsy following first febrile seizure in Saudi children Ann Saudi Med 2017 37 06 449 454 29229893 10.5144/0256-4947.2017.449 PMC6074123

[17] Kira R Ishizaki Y Torisu H Genetic susceptibility to febrile seizures: case-control association studies Brain Dev 2010 32 01 57 63 19854014 10.1016/j.braindev.2009.09.018

[18] Veisani Y Delpisheh A Sayehmiri K Familial history and recurrence of febrile seizures; a systematic review and meta-analysis Iran J Pediatr 2013 23 04 389 395 24427491 PMC3883367

[19] Gontko-Romanowska K Żaba Z Panieński P The assessment of laboratory parameters in children with fever and febrile seizures Brain Behav 2017 7 07 e00720 28729928 10.1002/brb3.720 PMC5516596

[20] Rivas-García A Ferrero-García-Loygorri C Carrascón González-Pinto L Mora-Capín A A Lorente-Romero J Vázquez-López P Simple and complex febrile seizures: is there such a difference?. Management and complications in an emergency department Neurologia 2022 37 05 317 324 10.1016/j.nrleng.2019.05.010 35672118

[21] Tang J Yan W Li Y Zhang B Gu Q Relationship between common viral upper respiratory tract infections and febrile seizures in children from Suzhou, China J Child Neurol 2014 29 10 1327 1332 24453149 10.1177/0883073813515074

